# Hydrophilic Stent Coating Inhibits Platelet Adhesion on Stent Surfaces: Initial Results In Vitro

**DOI:** 10.1007/s00270-018-2036-7

**Published:** 2018-07-23

**Authors:** Tim Lenz-Habijan, P. Bhogal, Marcus Peters, Albrecht Bufe, Rosa Martinez Moreno, Catrin Bannewitz, Hermann Monstadt, Hans Henkes

**Affiliations:** 1 phenox GmbH, Lise-Meitner-Allee 31, 44801 Bochum, Germany; 2 Neuroradiologische Klinik, Neurozentrum, Klinikum Stuttgart, Stuttgart, Germany; 3 Abteilung für Experimentelle Pneumologie, Medizinische Fakultät der Ruhr-Universität Bochum, Bochum, Germany; 4 Complejo Hospitalario Universitario Granada, Granada, Spain; 5 Medizinische Fakultät, Universität Duisburg-Essen, Essen, Germany

**Keywords:** Flow diverter stent, Anti-thrombogenic coating, Anti-platelet

## Abstract

**Background:**

Endovascular stents and flow diverter stents (FDS) have revolutionized the treatment of intradural aneurysms; however, the need for dual anti-platelet treatment (DAPT) limits their use and can cause additional issues. Therefore, there is a need to develop stent coatings that negate the need for DAPT.

**Methods:**

Two different hydrophilic polymer coatings (HPC-I and HPC-II) were used to coat small nickel titanium plates to initially test the hydrophilic properties of these coatings when applied to nickel titanium. The plates were subsequently incubated with non-medicated whole blood from healthy volunteers for 10 min and stained with a CD61 immunofluorescent antibody that allows detection of adherent platelets. The coatings were applied to FDS wires and were again incubated with non-medicated whole blood from the same volunteers. Scanning electron microscopy was used to detect adherent platelets on the wire surface.

**Results:**

The HPC-II coating (1.12 ± 0.4%) showed a significantly lower CD61 +ve cell count (*p* ≤ 0.001) compared to both uncoated NiTi plates (48.61 ± 7.3%) and those with the HPC-I coating (mean 40.19 ± 8.9%). Minimal adherent platelets were seen on the FDS nickel titanium wires coated with the HPC-II compared to uncoated FDS under electron microscopy.

**Conclusion:**

There is a significant decrease in the number of adherent CD61 +ve platelets on nickel titanium surfaces coated with the HPC-II coating compared to uncoated surfaces. The coating can be successfully applied to the wires of flow diverters. The results of this study are promising with regard to the development of new anti-thrombogenic endovascular devices.

## Introduction

During the last decade, we have witnessed the emergence of flow diverting stents (FDS) that are designed to treat intracranial aneurysms without the adjunctive implantation of coils. Although the exact mechanism by which flow diverters work remains elusive, it is thought the redirection of flow away from the aneurysmal sac and altered intra-aneurysmal flow with gradual thrombosis all play a role in the early stabilization of aneurysms with subsequent neo-endothelialization eventually remodelling the vessel wall and excluding aneurysm from the circulation [1]. These devices were originally evaluated in otherwise untreatable aneurysms or after failure of other endovascular treatments [2, 3], but numerous single-centre studies and meta-analyses have also been published demonstrating the efficacy and safety profile of these devices [4–13]. To date, six devices are available (Pipeline, Medtronic; FRED, MicroVention; Silk, Balt Extrusion; Surpass/Streamline, Stryker; Derivo, Acandis; p64 Flow Modulation Device and p48 Flow Modulation Device, both phenox) with newer devices in development.

Although flow diverters have revolutionized the treatment of aneurysms, one major issue remains unsolved. Due to the thrombogenic surface, platelet function-inhibiting drugs are needed to prevent in-stent thrombosis. However, these drugs are not without clinical consequences. Since the beginning of stenting as an endovascular therapeutic technique, early thrombosis in stented arterial segments has been a major concern [14]. The overall rate in the early clinical experience was as high as 24%. They were initially treated by aggressive anti-aggregation regimens, which reduced the incidence of thrombotic events, but this was associated with an increased rate of haemorrhagic complications [15]. With time, anti-platelet therapy alone demonstrated superior clinical results compared to other anti-thrombotic treatments in several studies [15, 16]. Typically, the dual anti-platelet regime (DAPT) consists of aspirin and clopidogrel for at least 3 months with continuation of aspirin for life thereafter. Naturally, there are issues regarding an increased bleeding risk and the ‘holy grail’ of endovascular implants is haemo-compatibility that does not require anti-platelet agents. Recently, the Pipeline Shield (Medtronic) has entered the market. This device, the first FDS with a thrombo-resistant coating, has a 3 nm thick covalently bound phosphorylcholine surface modification. Phosphorylcholine is a major component of the outer membrane of erythrocytes and has demonstrated efficacy in resisting platelet adhesion as well as intimal hyperplasia [17–19]. However, at the moment there are limited clinical data available on the use of this technology.

It is imperative to identify other potential surface coatings or modifications that may reduce thrombogenicity of stents. The purpose of this study was to develop and evaluate two new anti-thrombogenic stent coatings. The best coating was then tested on two FDS—the p64 and the p48 (phenox GmBH, Bochum, Germany).

## Materials and Methods

### Specimens and Coatings

Small nickel titanium plates (9 × 9 × 0.5 mm) and flow diverters served as substrate for the coating. The nickel titanium plates were laser-cut out of 0.5 mm thin nickel titanium sheets. In order to remove contaminations from the laser cutting process and to ensure a homogeneous TiO_2_, all plates were electrolytically polished and passivated. Two different hydrophilic polymer coatings (HPC-I and HPC-II) were applied on the specimens. HPC-I is a well-known polyethylene glycol (PEG)-based coating. HPC-II is a newly developed glycan-based multilayer polymer coating.

### Hydrophilic Testing

Small droplets of water are applied on the uncoated nickel titanium plates and uncoated FDS using a micropipette (Eppendorf^®^ Research^®^ plus; 2–20 μL) with 20 µL applied to the nickel titanium plates and 2 µL applied to the braided flow diverter stents. Changes in the hydrophilic properties of the coated samples were used to monitor coating efficiency.

### Blood Donors

Blood was voluntarily collected from eight healthy donors under the approval of the ethic commission of the Faculty of Medicine of the Ruhr-Universität Bochum, Germany (registration number: 16-5991). Exclusion criteria for participation in the study were an abnormal blood count measured via haematology analyser (KX-21 N, Sysmex, Norderstedt, Germany) and the intake of drugs that act on blood clotting. The number of platelets in the blood was recorded for each donor (not shown).

Informed consent was obtained from all individual participants included in the study.

All procedures performed in studies involving human participants were in accordance with the ethical standards of the institutional and/or national research committee and with the 1964 Helsinki Declaration and its later amendments or comparable ethical standards.

### Blood Contact In Vitro

All steps were performed under sterile conditions. Heparinized venous blood was collected using the “S-Monovette Blood collection system” (Sarstedt, Germany) containing 16U heparin. Before blood contact, specimens were rinsed twice in phosphate-buffered saline (PBS). The specimens were then incubated in 6-well plates in 2 ml blood for 10 min under dynamic conditions on a platform shaker (speed level 4; Titramax 100, Heidolph Instruments, Schwabach, Germany). After incubation, specimens were rinsed in PBS in order to eliminate non-adherent cells and analysed.

### CD61 Immunohistochemistry

Formation of platelet aggregates is mediated by cell adhesion molecules like CD61. CD61 (integrin β-3) is a glycoprotein found on both activated and inactive platelets and is involved in cell adhesion, spreading and platelet aggregation during haemostasis. Integrin is activated via G-protein coupled signal transduction and then binds fibrinogen. Fibrinogen stabilizes the aggregation of activated platelets by fibrinogen bridges. CD61 is a part of the platelet glycoprotein receptor IIb/IIIa (αIIbβ3; CD41/CD61) [20]. CD41/CD61 is known to bind to a variety of proteins including fibrinogen, fibronectin, von Willebrand factor and vitronectin [20]. CD61-positive adherent platelets were stained using CD61-PE antibody (BD Pharmingen, Heidelberg, Germany) fluorescence staining. After incubation in whole blood, nickel titanium specimens were rinsed in PBS twice in order to remove non-adherent cells. The antibody was diluted 1:5 in PBS and added to the nickel titanium specimens. After 15-min incubation in the dark, nickel titanium specimens were rinsed in PBS twice, fixed with 1% paraformaldehyde (Sigma-Aldrich, Taufkirchen, Germany) in PBS and analysed using a fluorescence microscope (Olympus BX40, Olympus; Hamburg, Germany) equipped with a digital camera (Moticam 5, Motic, Wetzlar, Germany).

### Scanning Electron Microscopy (SEM)

SEM was performed on a LEO 1530 Gemini (Carl Zeiss AG, Jena, Germany) equipped with an ISIS EDX system (Oxford Instruments, Wiesbaden, Germany). For SEM analyses specimens were incubated in whole blood for 10 min, rinsed in PBS twice, fixed with 3.7% glutaraldehyde (Sigma-Aldrich, Taufkirchen, Germany) in PBS for 15 min, dehydrated by an ascending series of alcohols, dried for 24 h at room temperature and sputtered with gold (Edwards Sputter Coater S150B9, Edwards Limited, Crawley, UK). SEM analyses were performed at the Ruhr-Universität Bochum, Faculty of Mechanical Engineering, Department of Materials.

### Semi-quantitative Phase Analyses

The positive area of platelet staining (CD 61 +ve stained cells) was analysed using ImageJ software (National Institutes of Health, Bethesda, MD) within defined regions of interest, 4 per nickel titanium plate, which were maintained for all samples (0.06 mm^2^ each, total are 0.24 mm^2^). The results were reported as percentage of positively stained area, which was calculated by dividing the absolute positive area by the predefined area of the region of interest.

### Statistical Analysis

Data were analysed by the Kruskal–Wallis test. If a significant difference was found, treated groups were compared with the control group by using Dunn’s post-test. GraphPad Prism software (version 3.03; GraphPad Software Inc., La Jolla, CA) was used for the analysis. P values of less than 0.05 were considered statistically significant. We started by testing the HPC-I and HPC-II coatings at the same time and in comparison with uncoated specimens. After 5 experiments of the HPC-I and HPC-II coatings, the experiments with HPC-I were abandoned due to futility. Further, ten experiments were conducted with the HPC-II coating in comparison with uncoated specimens (Table 1).

**Table 1:** The total number of experiments performed for each coating

HPC-I	HPC-II	Bare
*n* = 5	*n* = 15	*n* = 15

## Results

### The HPC-I and the HPC-II Coatings Increase Hydrophilicity

**Fig. 1:**
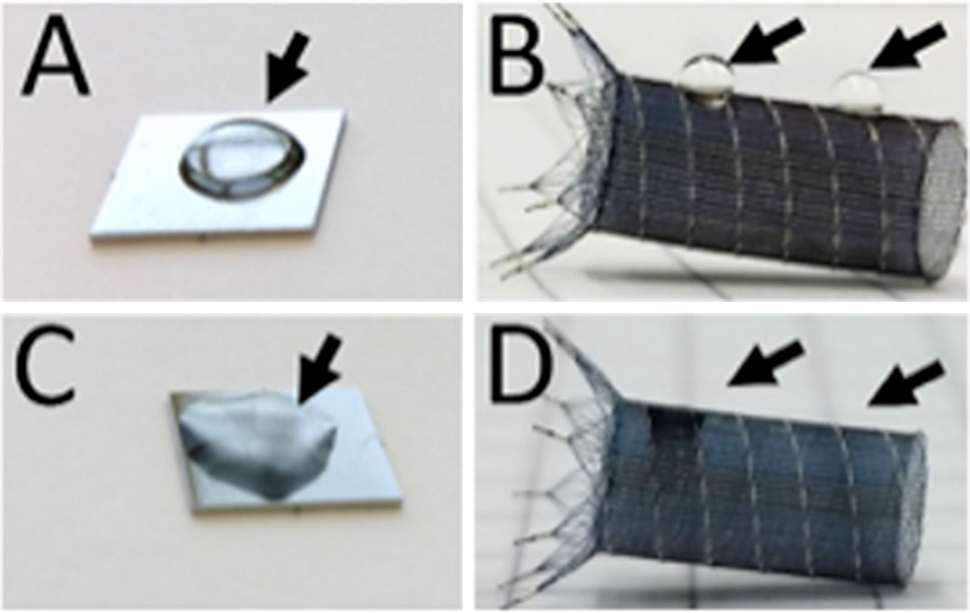
Comparison of the hydrophilic properties of uncoated (**A**, **B**) and hydrophilic-coated (**C**, **D**) nickel titanium plates and braided flow diverter stents. After application of H_2_O droplets on the uncoated specimens, the droplets remain in spherical shape on the surface of the specimens (**A**, **B**; arrows). Application of the same amount of water on the hydrophilic-coated specimens (HPC-II shown exemplarily) leads to a complete wetting of the sample (**C**, **D**)

Small droplets of water were applied onto the surface of the uncoated specimens. The droplets maintain a spherical shape on the surface of the nickel titanium plates (Fig. 1A, 20 µL) as well as on the braided flow diverter stents (Fig. 1B, 2 µL). Coating of the specimens results in an increased hydrophilicity. Application of the same amount of water on a coated specimen leads now to a breakdown in the surface tension of the water droplet and dispersion of the water over the surface of both the nickel titanium plate (Fig. 1C) and the flow diverter (Fig. 1D). The changes in the hydrophilic properties, which indicate the successful coating of the samples, were used to monitor the coating efficiency.

### The HPC-II Coating Significantly Reduces Adherence of CD61-Positive Platelets on Nickel Titanium Plates

**Fig. 2:**
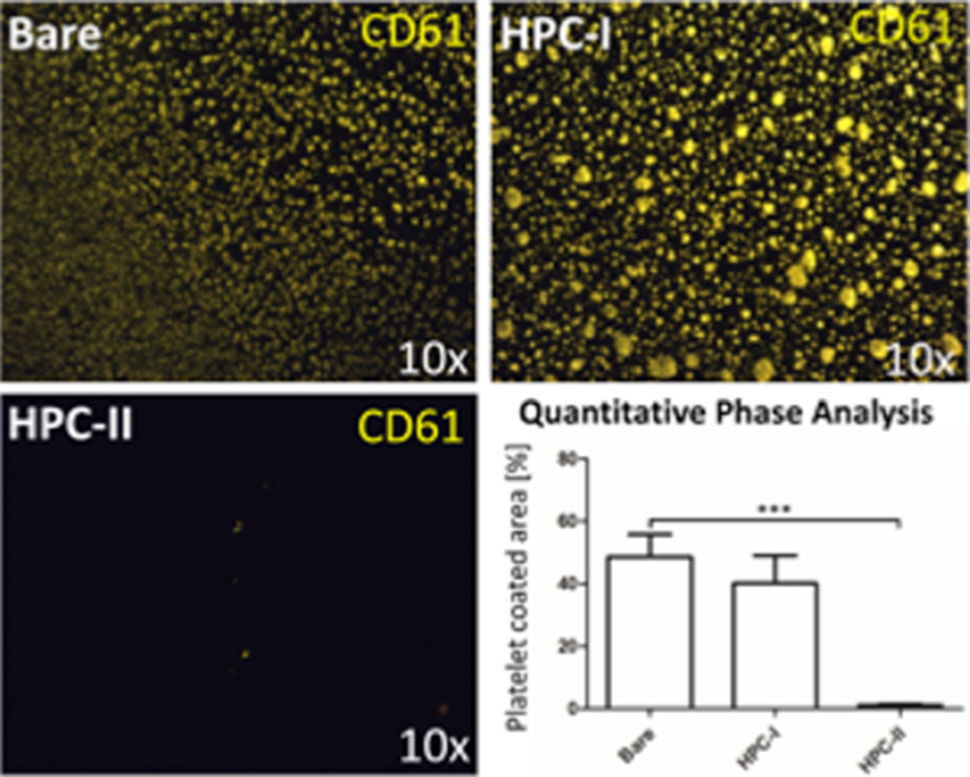
Representative fluorescence micrographs of uncoated (bare) and differently coated (HPC-I and HPC-II) nickel titanium specimens. The specimens were incubated in whole blood for 10 min under dynamic conditions. Adherent platelets were stained with a CD61 antibody (yellow fluorescence). Bottom right: Quantitative Phase Analysis of the area coated with CD61-positive cells from experiments with ten different donors (mean ± SEM, asterisks denote significance at *p* ≤ 0.05; ***p ≤ 0.001; Kruskal–Wallis and DUNN post-test; sample size HPC-I vs. bare, *n* = 5; sample size HPC-II vs. bare, *n* = 15)

The anti-thrombogenic properties of the HPC-I, HPC-II and uncoated nickel titanium plates were tested by incubating with whole blood, under dynamic conditions on a platform shaker, for 10 min. The blood, taken from healthy volunteers (*n* = 8), was non-medicated. Adherent platelets were visualized via a fluorescent CD61 antibody using a fluorescence microscope. Representative images are shown in Fig. 2. On the HPC-II-coated specimens very few CD61-positive cells were detected (Fig. 2). This effect was true for all blood donors. Quantitative Phase Analyses revealed a highly significant difference in the surface area covered in thrombocytes between the bare (48.61 ± 7.3%) and HPC-I (40.19 ± 8.9%) compared to the HPC-II-coated (1.12 ± 0.4%) nickel titanium plates.

### The HPC-II Coating Significantly Reduces Adherence of CD61-Positive Platelets When Applied to Two FDS of Different Design

The HPC-II coating was applied to two different flow diverters (p48 and p64, phenox, Germany). Both flow diverter stents were incubated in whole blood for 10 min under dynamic conditions on a platform shaker (speed level 4; Titramax 100, Heidolph Instruments, Schwabach, Germany).

**Fig. 3:**
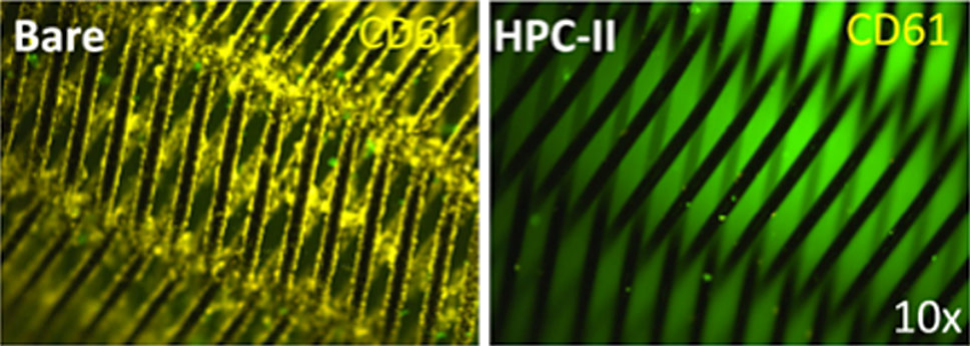
Representative fluorescence micrographs of uncoated (bare) and HPC-II-coated p64 flow diverter stents. The specimens were incubated in whole blood for 10 min under dynamic conditions. Adherent platelets were stained with a CD61 antibody (yellow fluorescence). The uncoated (bare) flow diverter stent is completely covered with adherent platelets, whereas only very few cells could be detected on the HPC-II-coated specimen. Again, HPC-II coating nearly completely prevents adherence of platelets even on the braided flow diverter stent

The uncoated (bare) specimens showed extensive coverage of the wires with adherent CD61 positive platelets and clumping of thrombocytes at the crossing points of the braided wires. The HPC-II-coated specimens exhibited a very low level of CD61 fluorescence (Fig. 3) with only sparse immunofluorescence and no clumping of thrombocytes. Due to their highly complex geometry, analysis of platelet adhesion and cell counts on flow diverters is difficult, and therefore, these analyses were restricted to the nickel titanium plates.

### Minimal Platelet Aggregation is Seen on HPC-II-Coated FDS on Electron Microscopic Examination

**Fig. 4:**
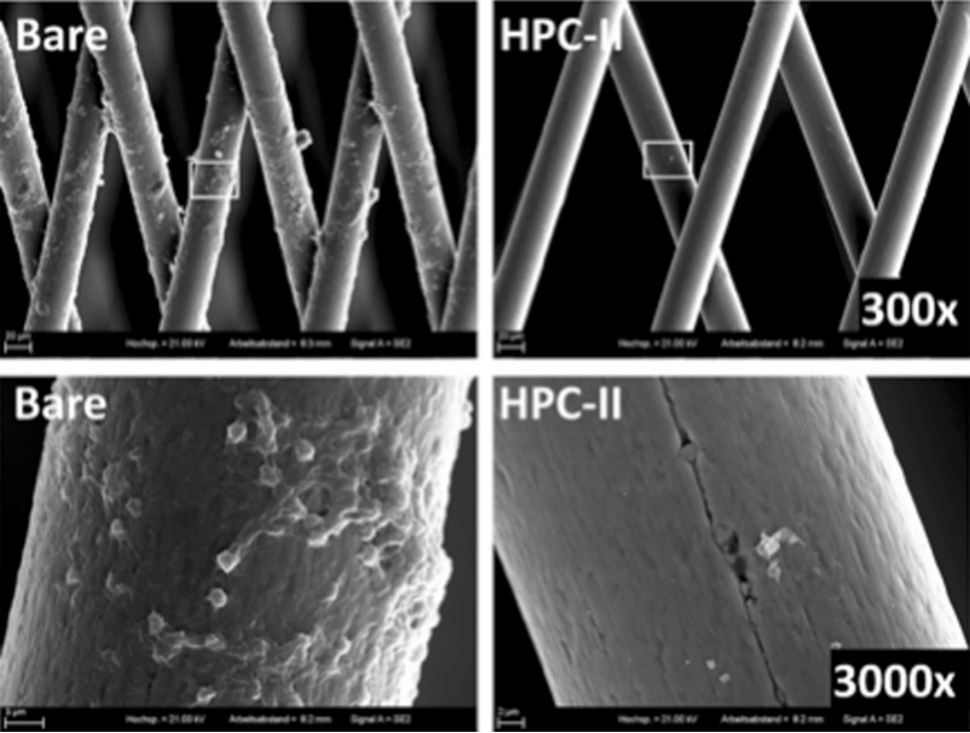
SEM micrographs of uncoated (bare) and HPC-II-coated p48 flow diverter stents. The specimens were incubated in whole blood for 10 min under dynamic conditions. Stents and adherent cells were fixed and sputtered with gold. The rectangle in the upper row indicates the area shown in a higher magnification below

In order to visualize the morphology of both the cells and the flow diverter surface, scanning electron microscope (SEM) pictures of coated and uncoated p48 flow diverter stents after incubation in whole blood for 10 min under dynamic conditions were taken. The micrographs are shown in Fig. 4. The uncoated stent shows a prominent layer of adherent cells (Fig. 4, bare). Cells adhere on the stent surface and on each other, forming small agglomerates (the preliminary stage of a blood clot) on and between the single wires of the stent. At higher magnification platelets on the uncoated flow diverter stent are diffusely spread over the wires of the FDS and they form a dense layer of cells. On the HPC-II-coated FDS, there are sparse cells adherent to the wires. Individual thrombocytes tended to adhere in the vicinity of defects on the wire surface (Fig. 4, HPC-II). Cell agglomerates were not detected.

## Discussion

Thrombosis on the surface of stents is complex, but protein adsorption seems to be the first step in the material–blood interaction, activating the intrinsic and extrinsic pathways of coagulation [21, 22]. The second step is platelet “rolling” and finally adhesion to the surface, or more accurately, to the adherent proteins on the surface. Eventually this process, together with different levels of trauma to the vessel wall related to the implantation of stents, may lead to the formation of platelet aggregates that progressively evolve into organized clots, and the activation of a local inflammatory response [21, 23, 24]. The results of our study showed a significant decrease in the number of CD61 +ve cells adherent to the HPC-II-coated nickel titanium plates as well as minimal thrombocytes adherent to the actual FDS with the HPC-II coating under electron microscopy. Given the complex geometry of flow diverters and the numerous contact points between the braided wires of the flow diverter, it is essential that any coating can be robustly applied to these wires. The ability to apply the HPC-II surface coating to nickel titanium wires, which can then be used to fabricate flow diverters, represents a significant step forward in the generation of devices that may not require dual anti-platelet use in clinical practice as well as minimizing the formation of clots on the surface of these devices. Furthermore, these results have shown that the coating is effective on two different flow diverter designs as both the p48, a flow diverter with 48 braided wires, and the p64, with 64 braided wires, were successfully coated. As these flow diverters are designed for different sized vessels, it is beneficial that the coating can be applied to both designs and raises hope that the coating could be applied to other intracranial stents other than flow diverters.

The development of clots on the surface of stents implanted in arterial structures could have significant clinical consequences, e.g. acute thromboembolic stroke. In order to prevent this from occurring, the standard approach to date has been to use anti-platelet agents that inhibit the formation of organized clots on the surface of the stents. The need for a stringent dual anti-platelet treatment (DAPT), typically involving aspirin and clopidogrel, prior to the implantation of stents has been advocated to reduce thromboembolic complications [25–27], and it is generally accepted that effective inhibition of platelet function can be achieved through DAPT [16, 28]. However, even with an optimized dosage of the anti-aggregant medication, the risk of thrombosis remains between 0.5 and 15% after stenting of the supra-aortic branches or vessels of the cerebrovascular circulation, being about seven times higher in intracranial vessels than in the coronary circulation [29, 30].

Despite numerous scientific efforts, especially in cardiology, it has not yet been possible to develop optimized anti-thrombogenic vascular implants. Regardless of a series of promising results in vitro [31–33], these coatings have not yet been successfully transferred to an endovascular device. Although the coating materials used to date for the coating of stents and flow diverters have bio- and haemo-compatible properties [32, 34], DAPT is still necessary. Approaches from other medical fields (e.g. the ProBIO^®^ coating of cardiovascular stents from Biotronik) or coatings used on catheters, contact lenses or in the field of molecular diagnostics in microarrays could offer new approaches for solving this problem. A transfer of such a coating to the highly complex geometry of flow diverters has not yet been shown successfully. Other anti-thrombogenic stent coatings (e.g. of the COBRA coronary stent, coated with polyzene-F) have anti-thrombogenic properties, but only shorten the period during which DAPT is required [35]. This may reduce the haemorrhagic risk of “responders”, but the management of “non-responders” and patients with acute haemorrhagic stroke is not facilitated by these coating concepts.

The development of devices that would minimize, or completely negate, the need for anti-platelet medication would represent a major leap forward. These devices would potentially limit the risks of both thrombogenic and haemorrhagic complications. Currently, the Pipeline Shield (Medtronic) is undergoing early-stage trials. This device has shown promising early results with 1.72 times fewer clots seen compared to the original pipeline device in one animal study [36] and similar results in an ex vivo model [37]. Several case reports have been published on the use of this device in patients with both ruptured and unruptured aneurysms [38–40]. In two of these cases there were no thromboembolic complications; however, in the case of Hanel et al. [39] the stent construct was thrombosed at the 10-day follow-up angiography and anti-platelet testing done at this time revealed an inadequate response to the maintenance dose of aspirin (81 mg). More recently, the early results from a prospective study using the Pipeline Flex with Shield technology have been published [41]. Fifty patients were enrolled in this prospective, single-arm, multicentre study of unruptured aneurysms. Anti-platelet testing was not mandatory. The vast majority of patients, 88% of those enrolled (44/50), received DAPT prior to treatment, and post-operatively all patients were on DAPT. In the 30-day post-operative period, there were 5 non-serious adverse events and 3 serious adverse events (SAE) as adjudicated by the clinical events committee (CEC), none of which were related to in-stent stenosis or acute intracranial haemorrhage. Unfortunately, it is still to be determined whether the Pipeline Shield can be used with mono-anti-aggregation.

The results of our study suggest the HPC-II coating is a promising coating that can minimize the thrombogenicity of nickel titanium. The HPC-II coating can be applied to nickel titanium wires which are used in the construction of flow diverter stents. The HPC-II coating has become “pHPC” as a part of the phenox brand name, and early clinical testing of flow diverters with this surface modification has begun with promising early results.

## Conclusion

This study demonstrates a significant decrease in the number of adherent CD61 +ve platelets on nickel titanium surfaces coated with the HPC-II coating compared to uncoated surfaces. The coating can be successfully applied to the wires of flow diverters where minimal adherent platelets were seen under electron microscopy. The results of this study are promising with regard to the development of new anti-thrombogenic endovascular devices. The HPC-I and HPC-II were working titles. HPC-II has become “pHPC” as a part of the phenox brand name.
